# Bispecific antibody targets and therapies in multiple myeloma

**DOI:** 10.3389/fimmu.2024.1424925

**Published:** 2024-10-10

**Authors:** Matthew Rees, Nadine Abdallah, Binoy Yohannan, Wilson I. Gonsalves

**Affiliations:** ^1^ Division of Hematology, Mayo Clinic, Rochester, MN, United States; ^2^ Division of Hematology, St Vincent’s Hospital Melbourne, Melbourne, VIC, Australia; ^3^ Division of Hematology, Oncology and Transplantation, University of Minnesota, Minneapolis, MN, United States

**Keywords:** bispecific antibodies, multiple myeloma, T-cell engagers, immunotherapy, combination (combined) therapy, sequencing, treatment resistance

## Abstract

Recently, several bispecific antibodies (BsAbs) have been approved for the treatment of relapsed multiple myeloma (MM) after early phase trials in heavily pre-treated patients demonstrated high response rates and impressive progression-free survival with monotherapy. These BsAbs provide crucial treatment options for relapsed patients and challenging decisions for clinicians. Evidence on the optimal patient population, treatment sequence, and duration of these therapeutics is unknown and subject to active investigation. While rates of cytokine release syndrome and neurotoxicity appear to be lower with BsAbs than with CAR T-cells, morbidity from infection is high and novel pathways of treatment resistance arise from the longitudinal selection pressure of chronic BsAb therapy. Lastly, a wealth of novel T-cell engagers with unique antibody-structures and antigenic targets are under active investigation with promising early outcome data. In this review, we examine the mechanism of action, therapeutic targets, combinational approaches, sequencing and mechanisms of disease relapse for BsAbs in MM.

## Introduction

Multiple myeloma (MM) is the second most common hematological malignancy in the United States, with an estimated 35,730 new cases diagnosed each year (1). The past two decades have witnessed remarkable progress in the therapeutic paradigm of MM with the introduction of immunomodulatory drugs (IMiDs), proteasome inhibitors (PIs), and anti-CD38 antibodies (Abs) (2, 3). This has significantly improved the prognosis of patients with MM, as evidenced by an increase in the 5-year relative survival rate from 32% to 58% (1). Despite this, the majority of patients will ultimately relapse and require additional therapies. The availability of newer generation IMiDs and PIs, such as pomalidomide, carfilzomib, and ixazomib, has expanded the treatment options in the relapsed/refractory (R/R) setting. However, treatment effectiveness decreases with each successive line of treatment, and patients experience shorter remissions (4). In the absence of an effective standard regimen, managing patients exposed to one or more agents from each major drug class (PIs, IMiDs, anti-CD38 Abs) has been challenging (5). With traditional therapies, less than a third of these patients will achieve a response, and only a minority will achieve a very good partial response (VGPR) or better. Patients who are triple-class refractory have especially poor outcomes, with an estimated overall survival (OS) of 6 to 9 months (5). The need for effective therapies for these patient populations has driven the development of MM immunotherapies, among which CAR-T (6, 7) and bispecific antibodies (BsAbs) that serve as T-cell engagers (TCEs) have exhibited unprecedented responses in heavily pretreated patients with MM, including those with triple-class refractory disease (6–8).

In the past three years, two CAR-T (7, 8) and three TCE BsAb products (9–11) were FDA-approved for MM, with BsAb products reserved for patients with ≥4 prior lines of therapy. While both drug classes function by redirecting T-cells towards plasma cells, TCE BsAbs leverage the antitumor activity of endogenous T-cells and, therefore, do not require *ex vivo* engineering. Teclistamab, a B-cell maturation antigen (BCMA) targeting BsAb, was the first BsAb to receive accelerated FDA approval in 2022. Two additional BsAb products gained accelerated approval in 2023: elranatamab, a BCMA-targeting BsAb (10), and Talquetamab, which targets G protein–coupled receptor, family C, group 5, member D (GPRC5D) (11). Several other BsAbs constructs are currently under development, targeting BCMA, GPRC5D, and other MM antigens including Fc receptor-homolog 5 (FCRH5) and CD38. This review will discuss MM BsAbs, their mechanism of action, pivotal clinical trials leading to their approval, associated clinical challenges, and future perspectives on their role in MM.

## Mechanism of action

**Figure 1:**
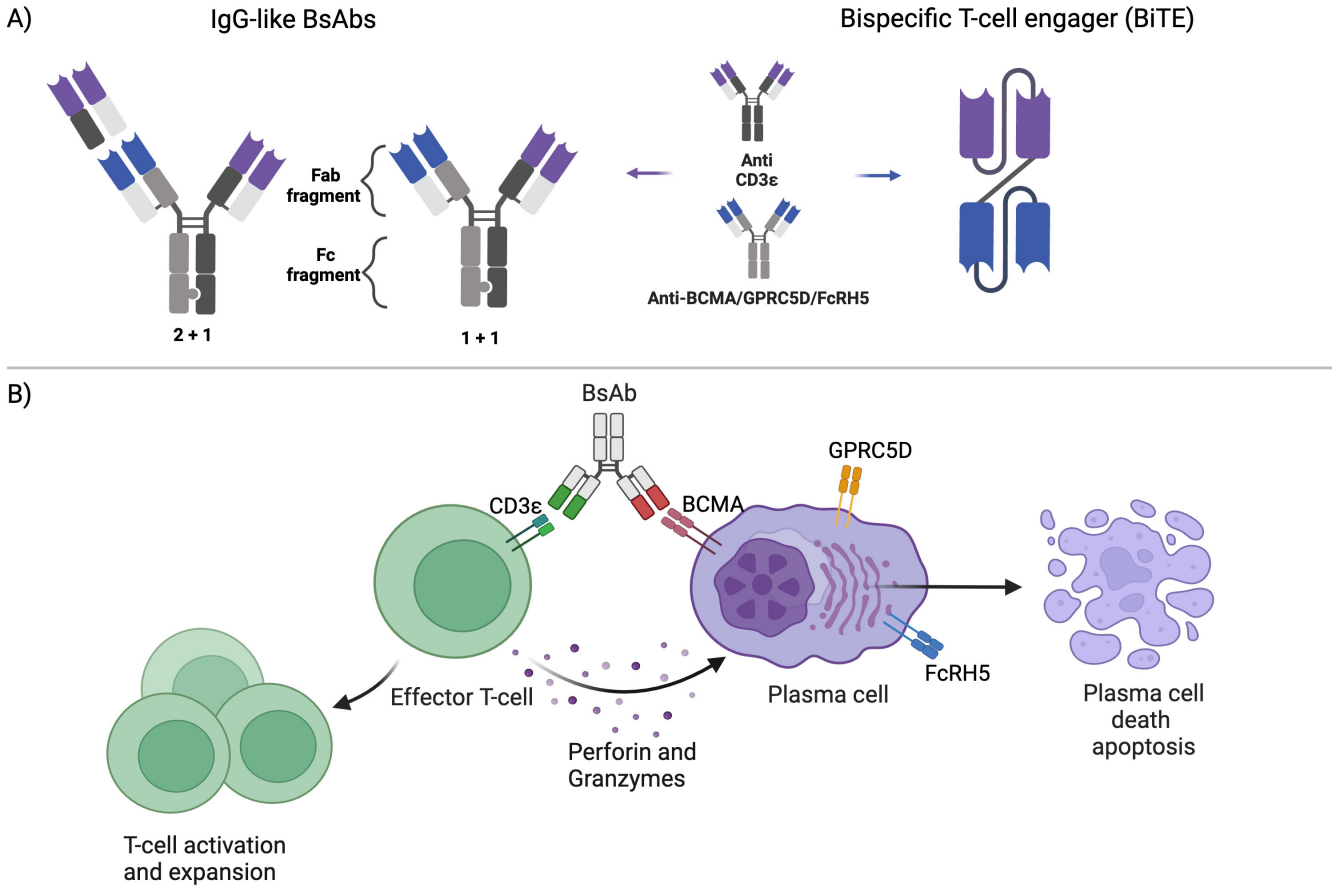
Bispecific antibody structure and function: **(A)** The main formats of bispecific antibodies (BsAbs) in multiple myeloma are the 1) bispecific T-cell engagers (BiTEs) (right) and 2) IgG-like BsAbs (left), which are composed of one (1 + 1) or two (1 + 2) binding sites for the target antigen: **(B)** BsAbs bind simultaneously to the CD3 receptor on T-cells and the target antigen on the plasma cell surface, bringing them close. This leads to T-cell activation and expansion and the release of perforin and granzymes, leading to plasma cell death.

BsAbs are a class of therapeutic agents derived from two or more parent antibodies (**Figure 1A**). In contrast to endogenous antibodies, where the two binding sites target one specific antigen (bivalent monospecific), BsAbs can bind two distinct antigens or epitopes (bivalent bispecific). TCE BsAbs engage T-cells and tumor cells, with one (or more) binding site (s) targeting a specific antigen expressed on the plasma cell surface, and another site targeting the CD3 subunit of the T-cell receptor on autologous T-cells. This dual binding facilitates the bridging of T-cells and tumor cells, triggering T-cell activation, the release of inflammatory cytokines, and the formation of an immunological synapse. Subsequent T-cell degranulation and release of perforin and granzyme B mediate the killing of target cells via apoptosis (**Figure 1B**) (12). The T-cell activation induced by BsAbs occurs independently of MHC restriction and without costimulation. Moreover, activation only occurs when the BsAb binds to the tumor-associated antigen and circumvents undesired and non-specific T-cell activation (12, 13). Generally, BsAbs are created by combining two heavy-light chains from distinct antibodies. This is achieved through one of three methods: 1) Fusion of two hybridoma cell lines to form a hybrid hybridoma or quadroma, which secretes a blend of hybrid immunoglobulins including the desired BsAb (14). 2) Chemical conjugation of two antibodies or Fab fragments with different antigen specificities (15). 3) As recombinant proteins using genetic engineering (16). Currently, the predominant approach for BsAb production is genetic recombination. Many BsAb constructs with distinct pharmacokinetic and pharmacodynamic profiles have been developed. The two major classes of TCE BsAbs of clinical relevance in MM are the bispecific T-cell engagers (BiTEs) and IgG-like bispecific antibodies (**Figure 1A**). BiTEs are small (about 55 kDa) proteins composed of two single-chain variable fragments (scFvs) connected by short linker peptide sequences. One scFvs is derived from an anti-CD3 antibody, while the other is derived from an antibody targeting a tumor-associated antigen (17). Due to their small size and absence of an Fc domain, BiTEs have a very short half-life of just a few hours. Consequently, they necessitate continuous infusion to sustain adequate levels in the circulation (18). Conversely, IgG-like bispecific Abs are Fc-containing engineered Abs that resemble classic immunoglobulins. However, they are composed of 2 different heavy chains (heterodimeric) derived from different antibodies (19). Various technologies were developed to enable correct heavy chain pairing, including knobs-into-holes technique (20), and correct light chain pairing, such as the CrossMab technology (21), IgG-like BsAbs have longer half-lives than BiTEs, given their larger size and Fc domain, and as a result, they are dosed intermittently. Fc-containing TCEs undergo modifications to inactivate their Fc domain to prevent non-specific effector functions, including antibody-dependent cell-mediated cytotoxicity (ADCC), complement-dependent cytotoxicity (CDC), and antibody‐dependent cellular phagocytosis (ADCP) (22). All currently approved TCEs in MM have a 1 + 1 design, with one binding site for the target antigen and another binding site for CD3; some BsAbs have a 2 + 1 design, with two binding sites for the target antigen; alnuctamab (23) and ABBV-383 (24, 25) are IgG-like BCMAxCD3 BsAb which bind BCMA with high affinity at two sites and bind CD3 with low-affinity; this design maximizes their efficacy while minimizing cytokine release by T-cells. Forimtamig is another BsAb with 2 + 1 design targeting GPRC5D (26). Trispecifics Abs like HPN-217 have 3 binding sites for BCMA, CD3, and albumin (27).

## Therapeutic targets for BsAbs

**Figure 2:**
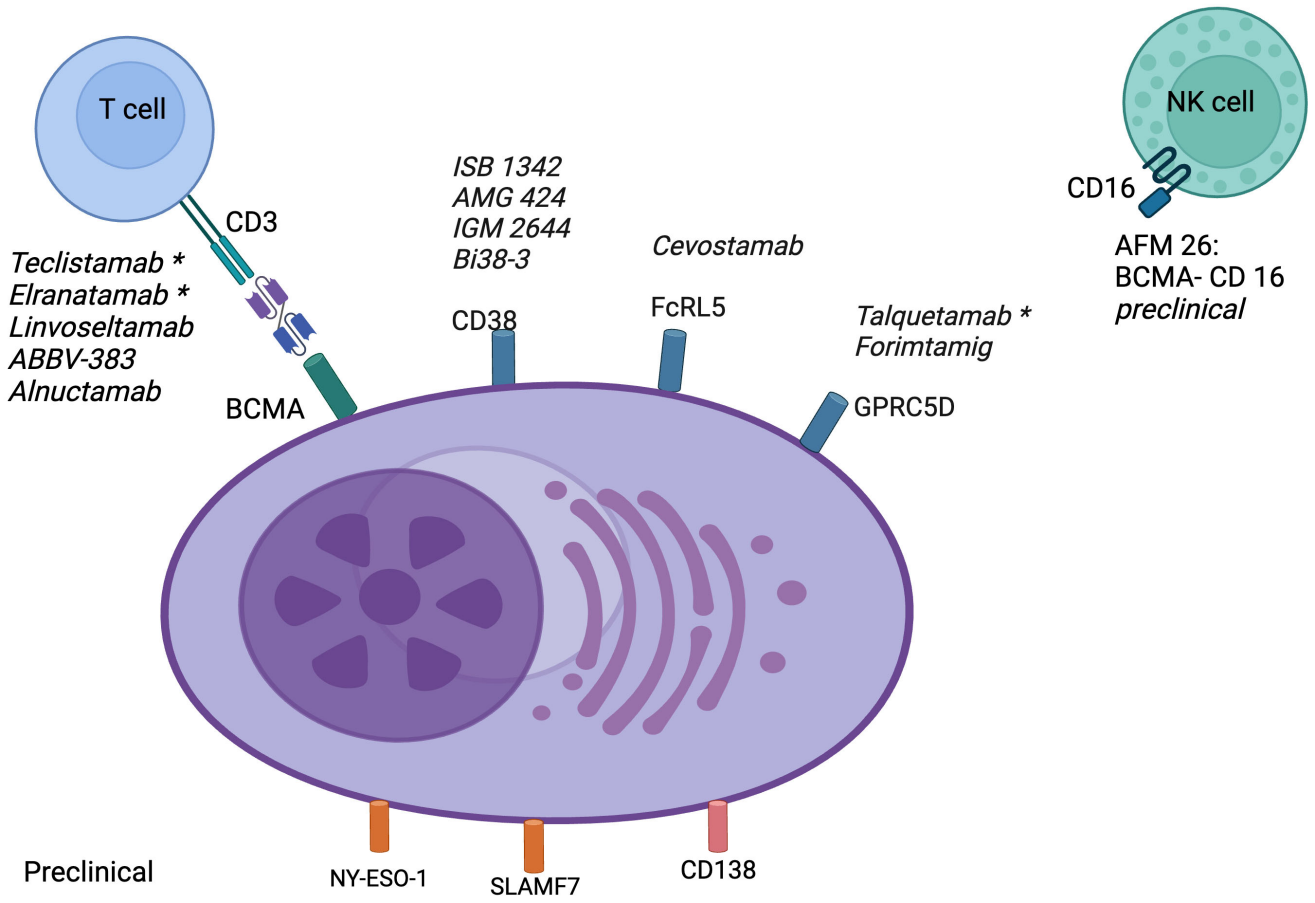
Targets for bispecific antibodies in multiple myeloma: BCMA, B-cell maturation antigen; CD, cluster of differentiation; Fc, fragment crystallizable; FcRL5, Fc receptor-like 5; GPRC5D, G protein-coupled receptor class C group 5 member D; NK, natural killer; NY-ESO-1, New York esophageal squamous cell carcinoma 1; SLAMF7, lymphocyte activation molecule family member 7. *FDA approved.

To maximize their tumor-specific activity and minimize on-target off-tumor toxicity, the ideal antigenic targets for BsAbs are those with high and uniform expression on the surface of target cells and no or minimal expression on normal cells. **Figure 2** summarizes the important therapeutic targets for BsAbs and drugs in various stages of development.

### BCMA-targeting BsAbs

1) BCMA, also known as TNFRSF17 or CD269, is a type III transmembrane protein that is selectively expressed on malignant plasma cells and critical for their survival, making it an ideal therapeutic target in MM (28). Multiple BCMA-targeted therapies with unique mechanisms of action have demonstrated great promise in R/R MM. The BsAbs have dual antigen specificity and bind to BCMA on the malignant plasma cells, and CD3 is expressed on the immune effector T cells, leading to T cell activation and tumor cell killing (29). The initial proof of concept for a TCE BCMA targeted BsAbs was provided by AMG 420, which demonstrated impressive clinical activity with durable remissions in R/R MM patients. CRS occurred in 38% of patients and there was no neurotoxicity events reported. Further development of this promising drug was halted due to its short half-life and need for continuous intravenous infusions (18).

There are currently two FDA-approved BCMAs targeting bispecific antibodies.

- Teclistamab was the first TCE BsAbs approved by the FDA in patients who had received four or more prior lines of therapy, including a CD 38 Ab, PI, and IMiDs. The dose escalation study identified 1.5 mg/kg as the recommended phase 2 dose (RP2D) with two step-up doses to mitigate cytokine release syndrome (CRS) risk. In the pivotal phase 1/2 MajesTEC-1 study, 165 patients (median of 5 prior lines of therapy, triple class refractory=77.6%) received the RP2D of teclistamab, and it showed an impressive overall response rate (ORR) of 63%, ≥ VGPR of 58.8% and 39.4% having ≥ complete response (CR). Among patients with ≥ CR, 30 (46%) achieved measurable residual disease (MRD) negativity. The median progression-free survival (PFS) was 11.3 months, and the median duration of response (DOR) was 18.4 months. CRS occurred in 72% of patients (grade 3, 0.6%). The subgroup analysis of the MajesTEC-1 study showed that the response rates were lower in patients with high disease burden (bone marrow plasma cells >60%, extramedullary disease, and R-ISS stage III); however, the response rate of patients with standard and high-risk cytogenetics was comparable. Infections were common and an important source of morbidity, with 44.8% having ≥ grade 3 or 4 infections (9).
- Elranatamab was the second FDA-approved CD3/BCMA BsAb (30). In the phase 1 MagnetisMM-1 trial, elranatamab showed promising safety and efficacy in R/R MM (31–34). The phase 2 registration study (MagnetisMM-3) enrolled 123 heavily pretreated MM patients (median of 5 prior lines of therapy, triple class refractory= 96.7%, penta-drug refractory=42.3%) ORR was 61% with 35% achieving ≥ CR and ≥ VGPR in 56.1%. Among MRD evaluable patients who achieved ≥CR, 89.7% achieved MRD negativity. The median PFS and DOR were not reached. The PFS rate at 15 months was 50.9%. CRS occurred in 56.3% of patients with no grade 3 or higher events. Like teclistamab, patients with a high bone marrow plasma cell burden (>50-60%) had a lower ORR with elranatamab. Infections were seen in 69.9% of patients; amongst them, 39.8% had ≥ grade 3, and 6.5% succumbed to their infections (10).
- Linvoseltamab: Investigational TCE BsAbs that has shown encouraging clinical activity in R/R MM (35). In a phase I/II study, among patients who received the 50 mg dose (n=104), the ORR was 48.1% with 21.2% achieving ≥ CR. In the 200 mg cohort (n=117), the ORR was 71% with 50% achieving ≥ CR and the median DOR was 29.4 months. In patients who had prior exposure to a BCMA directed antibody drug conjugate (ADC) the ORR was 70%. CRS occurred in 46% of patients (≥ grade 3=0.9%) treated at the 200 mg dose and infections were seen in 74.4% of patients (36).
- Other investigational BCMA targeting agents:
  i. Alnuctamab is a 2 + 1 TCE BsAbs with dual BCMA binding domain. In a phase 1 study (NCT03486067), the ORR was 83.3% in the intravenous alnuctamab cohort (≥ 6 mg); however, CRS occurred in 89.5% of patients (37). In the subcutaneous cohort (n=41), the target 30mg dose produced an ORR of 69% (≥CR = 43%) and the median PFS was not reached at a median follow up of 9.3 months (38). Bristol Myers Squibb withdrew development of Alnuctamab in May 2024 citing a change in business objectives.
  ii. ABBV-383 is a TCE BsAbs targeting BCMA. Preliminary results from a phase I trial identified an ORR of 68%, with estimated 12-month PFS of 58% for doses ≥40mg (24, 25). A phase 3 clinical trial (NCT06158841) is currently enrolling to assess the activity of ABBV-383 versus standard available therapies in R/R MM.
  iii. HPN-217 is a novel trispecific construct with three binding domains, including anti CD3 for T cell engagement, anti-BCMA for binding to plasma cells and anti-albumin domain for half-life extension (27).
  iv. WVT078 is a CD3/BCMA BsAb with a high affinity for BCMA and demonstrated potent T cell activation in preclinical studies. Early phase human studies have shown encouraging clinical activity (39). Investigational TCE BsAbs in MM are summarized in **Table 1**.

**Table 1:** Summary of investigational TCE BsAbs and trispecific constructs in MM clinical trials.

Drug	Target	Study	Population size (N)	Median prior lines of treatment	Triple class refractory (%)	Penta drug refractory (%)	ORR (%)	≥CR, (%)	Cytokine release syndrome (any grade/≥grade 3), %	Median PFS (months)	Median OS (months)
Linvoseltamab (35, 36)	BCMA/CD3	Linker-MM1	252	5 (1-16)	81	24	50 (50 mg) 71 (200 mg)	21 50	54.8/1.9 46.2/0.9	NR NR	NR NR
HPN-217 (27)	BCMA/CD3/Albumin	NCT04184050	62	6 (2-19)	76	42	51		28/1	NR	NR
ABBV-383 (24, 25)	BCMA/CD3	NCT03933735)	124	5 (3-15)	82	35	57	28	71/2	NR	NR
WVT078 (39)	BCMA/CD3	(NCT04123418)	33	5 (2-13)	51.5	18.2	38.5 (48–250 µg/kg) 70 (250 µg/kg)	11.5 (48–250 µg/kg)	60/12 75/25	NR	NR
Alnuctamab (37, 38)	2 + 1 BCMA/CD3	ALNUC; BMS-986349	IV cohort (n=19) SC cohort (n=73)	6 (3-12) 4 (3-14)	63	19	52.6 54	21.1 33	89.5/5.8 40/0	10.1	NR
Forimtamig (26)	GPRC5D×CD3	NCT04557150	*N* = 108 IV cohort (n=51). SC cohort (n=57)	5 (2-15) 4 (2-14)	62 72	36 42	71 64	35 26	82.4/2 78.9/1.9	NR NR	NR NR
Cevostomab (51)	FcRH5/CD3	NCT03275103	161	6 (2-18)	85	68	57 (132-198) mg dose level	8.4	83/1.6	NR	NR

IV, intravenous; s/c- subcutaneous; NR, not reported.

### Non-BCMA-targeting BsABs

#### GPRC5D targeting BsABs

GPRC5D is an orphan receptor expressed on keratinized tissues such as hair follicles, nail beds, and eccrine sweat glands in the skin (40–42). In addition, GPRC5D expression is higher in malignant plasma cells than in normal plasma cells and is associated with high-risk disease (40, 43–45).

- Talquetamab is the first-in-class bispecific IgG 4 antibody that binds to CD3 on T cells and GPRC5D on plasma cells, forming an immunological synapse and T cell-mediated killing of malignant plasma cells. In the pivotal phase 1 MonumenTAL-1 trial (n=232), patients with R/R MM (median of six prior lines of therapy), single agent talquetamab showed an impressive ORR of 64-70% with 52-60% ≥ VGPR. The ORR in the triple-class refractory in the 405-μg/kg and 800-μg/kg dose levels were 65% and 70%, respectively, whereas, in the penta-class refractory group, the ORR was 78% and 83%, respectively. In patients who received a prior BCMA BsAb or CAR-T cell product, the ORR was 50%. The median DOR at the 405-μg/kg dose level and 800-μg/kg dose level was 10.2 months and 7.8 months, respectively. CRS occurred in 77% (≥ grade3, 3%) of patients treated at 405-μg/kg dose and 80% (≥ grade3, 0%) of patients at the 800-μg/kg dose. Since GPRC5D is highly expressed in keratinized tissues, patients experience unique off-tumor, on-target adverse events such as skin desquamation, dysgeusia, and nail changes that adversely affect their quality of life (11). Lower intensity and less frequent dosing after achieving a response could be an important step to mitigate the toxicity while maintaining the efficacy of talquetamab (46).
- Forimtamig, also known as RG6234, is a dual-binding TCE BsAb with a unique 2:1 configuration that binds with high avidity to GPRC5D on malignant plasma cells and CD3 on T cells. Forimtamig has a silent Fc region that abrogates ADCC, ADCP and CDC related toxicity (26, 47). Forimtamig is believed to be more potent, given its 2:1 configuration and has shown significant cytotoxicity in preclinical models against all GPRC5D-positive MM cell lines (48). In the phase 1 dose escalation study, forimtamig showed impressive results with an ORR of 71.4% (≥ VGPR:57.1%) in the IV cohort vs 60.4% (≥ VGPR:39.6%) in the subcutaneous cohort (26). At a median follow-up of 11.6 months in the IV cohort, the median DOR was 10.8 months. Among patients who achieved a CR, 71.8% had MRD negativity. Pharmacodynamic data showed that there is delayed and lower CRS with subcutaneous administration (26).

#### FCRH5 targeting BsABs

FCRH5 is a cell surface marker that is expressed on mature B cells, including plasma cells, with a higher expression on malignant plasma cells (49–51).

a) Cevostamab is a TCE BsAb that binds to CD3 on T cells and FcRH5 on malignant plasma cells, resulting in potent killing of MM cells. In a phase 1 study (n= 160 patients, 85% triple class refractory, ≥1 prior CAR-T= 28, 17.5%, ≥1 prior anti-BCMA therapy=33.8%), single-agent cevostomab demonstrated meaningful clinical activity with an ORR of 54.5% at the 160 mg dose level. The responses were quite durable even in patients who had prior therapy with a BCMA targeting BsAbs, antibody-drug conjugate, or CAR-T cell therapy (52). In the subset of patients (N= 18) who discontinued therapy after 17 cycles, more than two-thirds maintained the response at a median follow-up of 9.6 months. The most common treatment emergent adverse events included infections, hematological toxicity, and CRS (53). Patients who received double step-up dosing had a significantly lower risk of CRS than those with single step-up dosing (52). Prophylaxis with IL6 blocking antibody has been shown to significantly reduce the risk of CRS without compromising efficacy (54). The phase I/II CAMMA-2 study is evaluating the safety and efficacy of cevostamab in patients with triple-class refractory MM who had prior BCMA-targeted therapy. Among 21 patients (prior BCMA ADC=10, CAR-T=11) enrolled in the CAMMA-2 study, the ORR was 67% with 38% achieving ≥ VGPR (55, 56). Cevostamab is also being studied as a consolidation strategy after BCMA CAR-T cell therapy to rejuvenate the persisting CAR-T cells and activate the endogenous T cell against FCRH5. The phase 2 STEM trial is currently enrolling with the sequential T cell engagement approach targeting BCMA with CAR-T followed by FCRH5 bispecific consolidation (57). Targeting two different tumor antigens may help achieve a deeper response.

#### CD 3/CD38 BsABs

CD 38 is highly expressed in plasma cells and is the therapeutic target for daratumumab and isatuximab. Below is a summary of the CD 3/CD38 BsAb in various stages of development.

- AMG 424, a CD3/CD 38 BsAb, was shown to trigger T cell proliferation and kill plasma cells with both low and high CD 38 expression. In mouse models, AMG 424 inhibited tumor growth without significantly releasing cytokines (58). Unfortunately, further clinical development of AMG 424 was halted.
- ISB 1342 is a high-affinity BsAb that binds CD38 on malignant plasma cells and CD3ϵ on T cells. The CD38 binding epitope is different from that of daratumumab, and hence, *in vitro* studies showed that ISB 1342 induced apoptosis in MM cell lines, even in those with a lower sensitivity to daratumumab. In mouse models, ISB 1342 demonstrated good tumor control and toxicity, which was acceptable in cynomolgus monkeys (59). Preliminary results from the phase 1 dose escalation study of ISB 1342 showed that the treatment was well tolerated with moderate CRS (17%) (60). Overall, ISB 1342 appears to be a safe, effective, and promising CD3/CD38 BsAb; even in daratumumab refractory patients, larger studies to validate these results are awaited.
- IGM-2644 is a CD3/CD38 IgM BsAb with a single CD3 binding domain and ten binding sites for human CD38. In addition, it exhibits more potent T cell-dependent cellular cytotoxicity and complement-dependent cytotoxicity than isatuximab and daratumumab. Preclinical studies showed a lower risk of CRS and a better safety profile than other CD3/CD38 bispecific antibodies. When compared to bispecific IgGs, IGM-2644 has shown lower T cell fratricide (61). A phase 1 clinical trial (NCT05908396) is currently enrolling to evaluate the activity and safety of IGM-2644 in R/R MM.
- Bi38-3, a novel CD3/CD38 BsAb with two scFvs. Bi38-3 mediates T cell-mediated lysis of malignant plasma cells with high CD 38 expression and spares other cells with low or intermediate CD 38 expression. It is believed to be effective in daratumumab refractory patients, given the binding epitope on CD38 is different (62). Interestingly, in contrast to AMG424, Bi38-3 has less ‘off tumor’ effects, and induced no B, T and NK cells toxicity *in vitro*. The reason for this is unclear, but presumably relates to the avidity of TCE binding – as there was selective killing of cells with high levels of CD38, with no or limited toxicity against cells expressing intermediate levels of CD38, such as B, T or NK cells.
- Y150 is an asymmetric IgG-like BsAb that binds to CD3 and CD 38 (63). It has a lower affinity for CD3 to enhance safety. A phase 1 study (NCT05011097) dose escalation study is currently enrolling patients with R/R MM.

#### BsABs targeting antigens others than BCMA, GPRCGD, FCRH5 and CD 38

- CD138xCD3: CD 138 is highly expressed in plasma cells, making it a good therapeutic target. In MM RPMI-8226 cell lines, CD3/CD138 BsAbs showed potent T-cell activation and cytotoxicity (64).
- CD3xILT3 BsAb: Immunoglobulin-like transcript 3 (ILT3), a tyrosine-based inhibition motif-containing receptor, is highly expressed in malignant plasma cells and associated with poor prognosis. In mice models, it has been shown that CD3x ILT3 I BsAbs could induce T cell-dependent cytotoxicity, lower tumor burden, and prolong survival (65). ILT3 appears to be a promising therapeutic target being explored, and human clinical trials are under development.
- LAVA-051: CD1d is a class I major histocompatibility complex-like molecule that can be expressed by various myeloid, lymphoid, and plasma cell disorders. In MM, CD1d is highly expressed in the early stages of the disease; however, CD1d expression is lost with disease progression. LAVA-051 is a novel gamma delta BsAb that engages the Vd2-T cell receptor chain of Vg9Vd2-T cells and CD1d, thereby mediating the killing of CD1d-expressing malignant plasma cells by type 1 natural killer T cells. A phase 1/2a clinical trial (NCT04887259) showed that LAVA-051 was well tolerated in dose escalation. The company discontinued further clinical development of LAVA-051 (66).
- anti-CD3 × anti-SLAMF7 BsAb: Signaling lymphocyte activation molecular family 7 (SLAMF7) is an important marker of normal and malignant plasma cells and is a therapeutic target for elotuzumab. A phase I clinical trial is currently investigating a CD3 × SLAMF7 T cell engager with peripheral blood mononuclear cells in R/R MM (NCT04864522).
- NY-ESO-1xCD3: NY-ESO-1 is a cancer antigen highly expressed in multiple tumor types, including MM, and is often associated with poor outcomes. *In vitro* and *in vivo* studies have shown that a bispecific construct targeting CD3 and NY-ESO-1 is capable of causing lysis of MM cells (67).

## Combination therapy with BsAbs

Although BsAbs have demonstrated high efficacy as monotherapy, some patients do not achieve adequate responses and many others progress despite an initial response. Thus, BsAbs are currently being evaluated in various combinations with standard-of-care agents which have non-overlapping mechanisms of action and toxicity profiles. In addition, BsAbs targeting different antigens are being studied in combination, with the expectation that dual targeting would result in better and more durable responses, particularly in patients where the disease has been resistant to standard therapies like those with extramedullary disease.

1. Combination of BsAbs and daratumumab: The efficacy and safety of daratumumab-based combinations in upfront and R/R settings prompted the evaluation of daratumumab as a potential partner to BsAbs. A preclinical study elegantly demonstrated that daratumumab augments the activity of teclistamab through both immunomodulatory and direct antitumor effects. Specifically, the ex vivo activity of teclistamab was enhanced in bone marrow aspirates from patients pre-treated with daratumumab, as well as when added to MM cell lines in the presence of peripheral blood mononuclear cells from daratumumab-pretreated patients. This enhancement was attributed to the immunomodulatory effect of daratumumab, which depletes CD38+ regulatory T and B cells and creates a permissive environment for teclistamab. Co-treatment with daratumumab also led to increased teclistamab activity through direct antitumor effects (68). These findings provided a strong rationale for the multicohort phase 1/2 TRIMM2 (NCT04108195) study evaluating each of teclistamab and talquetamab in combination with daratumumab in patients with R/R MM who have had ≥3 prior lines of therapy; initial results have been reported for both talquetamab in addition to daratumumab and teclistamab in addition to daratumumab cohorts. After a median follow-up of 11.5 months, 65 patients received the combination, including 77% refractory to anti-CD38 Abs. The preliminary results were encouraging, with 78% achieving a response, including 45% ≥CR. All patients without prior exposure to anti-CD38 Abs achieved a response, compared to 76% for patients refractory to anti-CD38 Abs. Responses were durable at one year in 86% of responders (69). CRS occurred in 78% of patients (all grade 1-2) and 63% had infections (≥ grade 3, 25%). Preliminary results from the teclistamab + daratumumab cohort yielded similar results; after a median follow-up of 7.2 months, ORR was 78% in 37 evaluable patients at all dose levels, including 73% with ≥VGPR and 24% with ≥CR. CRS occurred in 54.5% (all grade 1-2) and infections occurred in 51.5% ((≥ grade 3, 24.2%) of patients (70).

Based on these promising results, several ongoing phase 3 studies are underway. The MonumenTAL-3 study (NCT05455320) is exploring the combination of talquetamab plus daratumumab with or without pomalidomide versus daratumumab-pomalidomide-dexamethasone in patients who had ≥1 prior line of therapy. Also, MagnetisMM-5 (NCT05020236) is evaluating elranatamab +/- daratumumab in patients with R/R MM (71).

1. Combination of BsAbs and IMiDs: Like daratumumab, preclinical studies have demonstrated synergistic interactions between IMiDs and BsAbs; in the presence of BsAbs, IMiDs exhibit a costimulatory effect, primarily mediated by the upregulation of IL-2 release (72, 73). This supported the clinical evaluation of BsAb + IMiD combinations, with or without anti-CD38 Abs. The ongoing multicohort phase 1b MajesTEC-2 trial evaluates teclistamab in various combinations in R/R MM patients. Initial data from 32 patients who received the triplet combination of teclistamab (0.72/1.5 mg/kg weekly), daratumumab, and lenalidomide (25 mg) showed promising results; after 8.4 months of follow-up, the ORR was 94% including 90% with ≥VGPR and 55% with CR. The combination was associated with a high infection rate (91%), mostly grade 1/2 (38% Grade 3-4) (74). This combination will be compared to daratumumab + lenalidomide + dexamethasone in transplant-ineligible newly diagnosed MM patients in the phase 3 MajesTEC-7 trial (NCT05552222). Talquetamab is also being evaluated in combination with various drug classes, including IMiDs in MonumenTAL-2 (NCT: NCT05050097). In a recent report, after 11 months of follow-up, 35 patients had received talquetamab + pomalidomide; high response rates were seen with both weekly (ORR: 87%/≥CR: 60%) and biweekly (ORR: 83%/≥CR: 44%) dosing schedules of talquetamab. No unexpected toxicities were seen; however, approximately one-third of patients required dose/schedule modifications due to adverse events (75).
2. Combinations of BCMA- and GPRC5D- targeted BsAbs: The RedirecTT-1 study studied a dual BCMA and GPRC5D targeting approach with a combination of teclistamab and talquetamab (N=93; 79.6% triple-class refractory). The ORR was 86.6% across all the dose levels, with 40.2% achieving a CR or better and a median PFS of 20.9 months. It is important to highlight that patients with extramedullary disease (n=35) had an impressive response (ORR of 71.4%, with 21.4% achieving a CR or better) (76).
3. Combinations of BsAbs and checkpoint inhibitors: Preclinical studies have shown that concurrent checkpoint inhibition can enhance the efficacy of BsAbs (77). Talquetamab and PD-L1 inhibitors can enhance the cytotoxic properties of NK and T cells against MM cells (78, 90). The TRIMM-3 is an ongoing phase 1 study that is exploring the combination of talquetamab with a programmed cell death protein-1 (PD-1) inhibitor in patients with R/R MM (NCT05338775).
4. BsAbs plus Cereblon E3 ligase modulatory drugs (CELMoDs): Recent studies have shown that CELMoDs could potentially mitigate the BsAbs-induced CRS by inhibiting the secretion of proinflammatory cytokines and, in addition, could enhance the clinical activity of BsAbs (23, 79). In preclinical models, it has been shown that CELMoDs could lower the risk of antigen-negative relapse in patients treated with forimtamig (80).
5. BCMA-BsAbs plus gamma-secretase inhibitors: Preclinical studies have shown that gamma-secretase inhibitors are capable of reducing soluble BCMA (sBCMA) levels and increasing BCMA expression in plasma cells to enhance the efficacy of the BCMA BsAbs (81). In the MajesTEC-2 trial (N=28 patients), the combination of a gamma-secretase inhibitor (nirogacestat) with teclistamab was evaluated in a heavily pretreated patient population (median of four prior lines of therapy, triple-class refractory=71%). The ORR and CR rates were 74% and 52% respectively. The median DOR was not reached; in the responding patients, more than two-thirds maintained a response for ≥ 1 year (82). A summary of ongoing trials of BsAbs in combination is provided in **Table 2**.

**Table 2:** Ongoing trials of BsAbs in combination with other agents.

Trial	Phase	Population	Regimens	Primary outcome	Status
NCT05090566 MagnetisMM-4	1b/2	RRMM ≥3 LOT TCR	Arm A: Elra + Nirogacestat Arm B: Elra + Len+ Dex	DLT	Recruiting
NCT05020236 MagnetisMM-5	3	RRMM	Part 1 Safety Lead-In Dose Escalation: Elra + Dara Part 2 Arm A: Elra	Part 1: DLT Phase 2: PFS	Recruiting
			Part 2 Arm B: Elra + Dara		
			Part 2 Arm C: Dara + Pom + Dex		
NCT04108195 TRIMM-2	1b	RRMM ≥3 LOT	Part 1: Dose Escalation: Dara + Tec Dara + Talq Dara + Talq + Pom Dara + Tec + Pom	DLT	Active Not recruiting
			Part 2: Dose Expansion RP2D for treatment combinations in Part 1		
NCT05137054	1b	RRMM	9 arms: Linvoseltamab plus: Dara Car Len Bort Pom Isa Fianlimab Cemiplimab Nirogacestat	DLT	Recruiting
NCT04586426 RedirecTT-1	1/2	Parts 1&2 RRMM Part 3: RRMM TCE	Part 1: Dose Escalation: Tec + Talq +/- Dara	DLT ORR	Recruiting
			Part 2: Dose Expansion: Tec + Talq +/- Dara		
			Part 3: Phase 2: Tec + Talq		
NCT06215118 MagnetisMM-30	1b	RRMM	Part 1 Dose Escalation: Elra + Iberdomide	DLT	Recruiting
			Part 2 Dose Randomization: Elra + Iberdomide		
NCT05675449 MagnetisMM-20	1b	RRMM Part 1: 1-3 LOT Part 2: ≥3 LOT TCR	Part 1 Dose Escalation: Non-randomized Elra + Car + Dex	DLT	Recruiting
			Part 2A Dose Escalation Elra + Maplirpacept		
			Part 2B Dose Randomization Elra + Maplirpacept		
NCT05338775 TRIMM-3	1b	RRMM	Part 1: Dose Escalation: Talq + PD-1 inhibitor Tec + PD-1 inhibitor	AE%	Recruiting
			Part 2: Dose Expansion: Talq + PD-1 inhibitor Tec + PD-1 inhibitor		
NCT05020236 MagnetisMM-5	3	RRMM ≥1 LOT	Part 1 Safety Lead-In Dose Escalation: Elra + Dara	Par 1: DLT Part 2: PFS	Recruiting
			Part 2 Randomized Arm A: Elra Arm B: Elra + Dara Arm C: Dara + Pom + Dex		
NCT06152575 MagnetisMM-32		RRMM 1-4 LOT	Elra	PFS	Recruiting
	3		Investigator’s choice: Elo + Pom + Dex or Pom + Bort + Dex, or Car + Dex		
NCT05623020 MagnetisMM-6	3	TI NDMM	Part 1 Elra + Dara + Len	Part 1: DLT Part 2: PFS sustained MRD	Recruiting
			Part 2 (randomized) Arm A: Elra + Dara + Len Arm B: Dara + Len + Dex		
NCT04722146 MajesTEC-2	1b	RRMM	8 arms Tec plus: Dara + Pom Dara + Len + Bort Nirogacestat Len Dara + Len Dara + Len + Bort	AE%	Active, not recruiting
NCT05572229 IFM2021-01	2	TINDMM ≥65 years ECOG 0-2	Tec + Dara Tec + Len	≥VGPR after 4 cycles	Not yet recruiting
NCT05083169 MajesTEC-3	3	1-3 LOT	Arm A: Tec + Dara	PFS	Active, not recruiting
			Arm B: Dara + Pom + Dex or Dara + Bort + Dex		
NCT05552222 MajesTEC-7	3	NDMM TI or not intended for ASCT as initial therapy	Tec + Dara + Len	PFS	Recruiting
			Talq + Dara + Len		
			Dara + Len + Dex		
NCT05849610 GEM-TECTAL	2	NDMM High-Risk	Induction Dara-VRD Intensification Tec-Dara Maintenance Tec-Dara + early rescue intervention Tal-Dara	MRD-neg CR	Recruiting
NCT04910568 CAMMA 1	1b	RRMM	Arm A: Cevostamab	RP2D	Recruiting
			Arm B: Cevostamab + Pom + Dex		
			Arm C: Cevostamab + Dara + Dex		
NCT05583617 PLYCOM	1b	RRMM ≥3 LOT TCE	Cevostamab + Iberdomide	AE%	Recruiting
NCT05927571	1b	RRMM	Cevostamab + elranatamab	AE%	Recruiting
NCT05050097 MonumenTAL-2	1b	RRMM ≥2 LOT	4 arms Talq plus: Car Dara + Car Len Dara + Len Pom	AE%	Recruiting
NCT06100237 REVIVE	2	High-Risk SMM	Tec + Dara Talq + Dara	MRD-neg 10^-5^ after 12 cycles (as BR)	Recruiting
NCT06208150 MonumenTAL-6	3		Arm A: Talq + Pom	PFS	Recruiting
			Arm B: Talq + Tec		
			Arm C: Elo + Pom + Dex or Pom + Bort + Dex		
NCT05455320 MonumenTAL-3	3	RRMM ≥1 LOT	Arm A: Talq + Dara + Pom	PFS	Recruiting
			Arm B: Dara + Pom + Dex		
			Arm C: Talq + Dara		
NCT06163898	1b/2	RRMM	Alnuctamab + Mezigdomide + Dex	AE%	Recruiting
NCT06055075	1b/2	RRMM	Forimtamig + Car Forimtamig + Dara	AEs%	Recruiting
NCT05695508 MajesTEC-5	2	NDMM TE ≤70 years ECOG 0-2	Tec-DRd - ASCT - Tec-DR	AE%	Recruiting
			Tec-DVRd - ASCT - Tec-DR		

ASCT, autologous stem cell transplantation; Bort, bortezomib; Car, carfilzomib; Dara, daratumumab; Dex, dexamethasone; ECOG PS, eastern cooperative oncology group performance status; Elra, elranatamab; Isa, isatuximab; Len, lenalidomide; LOT, lines of therapy; Pom, pomalidomide; RRMM, relapsed refractory multiple myeloma; SMM, smoldering myeloma; Talq, talquetamab; TE, transplant-eligible; Tec,: teclistamab; TI, transplant-ineligible. Available from: https://clinicaltrials.gov/. Accessed: 08/12/2024

## Advantages and disadvantages of BsAbs over other cellular therapies

The advent of CAR T and BsAbs has created crucial treatment options for MM patients and challenging decisions for clinicians. Evidence on the optimal patient population, treatment sequence, and duration of these therapeutics is unknown and subject to active investigation. The application of these therapies is also governed by access, prior treatments, performance status, disease trajectory, and patient preference. **Table 3** summarizes key characteristics of FDA approved BsAbs and cellular therapies, including their strengths and weaknesses.

**Table 3:** Comparison of the key characteristics of FDA approved CAR T and bispecific antibodies.

Feature	Bispecific T cell Antibodies	CAR T cell therapy
**FDA approved agents, date of approval**	Teclistamab, October 2022 Elranatamab, August 2023 Talquetamab, August 2023	Ide-cel, March 2021 Cilta-cel, February 2022
**Hospitalization required**	Cycle 1	Yes
**Treatment frequency**	Q1-2 Weekly Until disease progression or intolerance	Once
**Manufacturing time**	Off the shelf	4 - 8 weeks (autologous)
**Specialized center required for delivery**	Yes, cycle 1	Yes
**Overall response rate, %**	60-70	75-95
**Median progression-free survival†** - **Relapsed multiple myeloma ≥ 3 prior lines of therapy**	11-15 months	9-35 months
**Cytokine release syndrome** **Grade all/≥3, %**	60-70/1	85-95/5
**Immune-cell effector associated neurotoxicity syndrome.** **Grade all/≥3, %**	3/0	5/1
**Infections** **Grade all/≥3, %**	BCMA: 70-75/40-45 GPRC5D: 35-40/5-10	BCMA: 60-65/25
**Hematological adverse events** **1. Neutropenia** **2. Anemia** **3. Thrombocytopenia Grade all//≥3, %**	1. Neutropenia: 40-70/30-70 2. Anemia: 50-65/40-50 3. Thrombocytopenia: 30-50/15-20	1. Neutropenia: 80-90/75-90 2. Anemia: 55-65/35-50 3. Thrombocytopenia: 54/41-42
**Hypogammaglobulinemia, %**	75-95	90
**Other toxicity**	Prolonged cytopenias Continuous therapy	Prolonged cytopenias
**Expense**	$$	$$$

ORR, PFS and toxicity data pertain to FDA approved agents used as monotherapy in the relapsed/refractory setting. † median PFS values based on extended follow up from phase 1-2 clinical trials (NCT03145181/NCT04557098, NCT04649359, NCT03399799, NCT02658929 and NCT03548207).

CAR T products, such as cilta-cel, have superior efficacy to BsAbs at the expense of increased toxicity. Additionally, CAR T access is hampered by manufacturing constraints, a limited number of accredited centers, and socioeconomic barriers (7–9, 31, 83). The frequency and severity of CRS and neurological toxicity are increased with CAR T compared to BsAb therapy (7–9, 11). While the timeline of these toxicities is predictable and pre-emptive strategies have improved safety (84), its management in medically frail patients and the potential for fatal complications such as immune effector cell-associated hemophagocytic lymphohistiocytosis-like syndrome (IEC-HS) remain problematic (85). Idiosyncratic CAR T toxicities also exist, following cilta-cel delayed onset of cranial nerve palsies, and neurocognitive adverse events (micrographia, tremors, inattention, psychomotor retardation) affect 5-10% of patients (8, 86).

Commercial CAR T manufacturing times range from 4-8 weeks, and stabilizing R/R MM patients over this period is challenging. In the KarMMa-3 and CARTITUDE-4 studies, 10% and 15% of patients allocated to the experimental arms did not receive the assigned intervention primarily because of disease progression before product availability (7, 8) ‘*Off-the-shelf*’ allogeneic products and rapid manufacturing protocols, such as the NEX-T process enable CAR T administration in under one week, obviating the need for bridging strategies (87, 88). The requirement for accreditation from the Foundation for the Accreditation of Cellular Therapy (FACT) in North America or the Joint Accreditation Committee ISCT-Europe & EBMT (JACIE) in Europe limits CAR T delivery to large academic centers with the necessary staff and budget. Such institutions are not evenly distributed within and between national borders. Furthermore, the cost of CAR T products remains prohibitively high for many government-funded health systems, and recipients experience numerous out-of-pocket costs secondary to transportation, accommodation, and caregiver demands.

In comparison, BsAbs offer an immediate, highly effective ‘*off-the-shelf’* therapy, filling a critical niche for relapsed patients with rapidly progressive disease. BsAbs also produces less frequent and severe immune effector cell toxicities, which is appealing for unfit and medically complicated patients. While all FDA-approved BsAbs require hospitalization for ramp-up, their ongoing administration can generally be in local community centers. BsAb therapy also shows a promising readiness to partner with other MM therapies, as discussed above, and these combinations may overcome differences in response rates and survival compared to CAR T (70, 75, 76).

The need for continuous treatment and the considerable cumulative infectious risk of BsAb therapy are important limitations of this therapeutic class. Time-off therapy is increasingly rare in the era of modern MM therapy but is associated with psychological and physical benefits as well as improved quality of life (89). Shifting from a weekly to a biweekly dosing schedule after achieving a durable response has been shown to maintain efficacy and alleviate treatment burden (90). Several investigational bispecific products utilize less frequent dosing schedules, such as ABBV-383 which is being administered on once per four-weekly basis from treatment initiation in its ongoing phase 3 clinical trial (NCT06158841). The phase I trial of cevostamab also suggests that time-limited therapy is feasible. Herein, cevostamab was administered for one year, with most patients who sustained a response to this point continuing in remission for at least 6-months from the end of therapy (53). Time-limited therapy for one year using the GPRC5D-directed bispecific antibody, forimtamig, has also shown promising efficacy with early results indicating a median DOR of 14 months (91).

While hematological toxicity, CRS, and ICANS rates are generally equivalent amongst BsAbs, the non-BCMA directed BsAbs, talquetamab, forimtamig, and cevostamab, result in a lower incidence of infections. This is likely because non-BCMA directed TCE BsAbs spare terminally differentiated B-lymphocytes, resulting in less B cell aplasia than BCMA-directed TCE BsAbs (11, 26, 52).

## Mechanism of resistance

### Disease refractoriness

Depending on the population treated, one half to a third of patients are refractory to BsAb therapy. Features associated with disease refractoriness include T-cell exhaustion, extramedullary disease (EMD), and high tumor burden. Teclistamab, elranatamab, and talquetamab BsAbs have all shown significantly lower ORRs in patients with EMD, 36%, 40%, and 46%, respectively. (9, 11, 31).

The *‘sink effect’* created by high levels of sBCMA, which acts as a decoy for BsAb binding, may partially explain the detrimental impact of disease burden and EMD on BCMA-directed therapy (9, 93, 105). Preclinical models have demonstrated that high sBCMA attenuates the binding of BCMA therapies (106, 107). Likewise, increased sBCMA levels correlate with lower response rates in clinical trials (35, 108). Partnering BCMA-directed BsAbs with other agents appears promising in overcoming this hurdle. Gamma-secretase inhibitors reduce sBCMA levels, improve BsAb cytotoxicity *in vitro* (81), and augment clinical response rates (82). Dual antigenic targeting to overcome BCMA attenuation, such as in the RedirecTT-1 trial with teclistamab and talquetamab, also appears effective (76). Nonetheless, EMD and disease burden also compromise non-BCMA BsAbs, indicating sBCMA-independent mechanisms are relevant, such as a low effector-to-target ratio and impaired T-cell infiltration of large lesions (11, 109).The extramedullary tumor milieu may also hamper immunotherapeutic approaches since immune checkpoint molecules, immunosuppressive cytokines [i.e., transforming growth factor-beta (TGF-β), and interleukin-10 (IL-10)], and immunosuppressive cells hinder the anti-tumor immune response and limit immune effector cell entry to these sites (110–112).

Correlative studies based on MajesTEC-1 and MagnestisMM-1 identify the T-cell landscape at treatment initiation as a major determinant of BsAb efficacy. Non-responders have features of a depleted immune system, namely, limited CD8+ naïve T-cells, a higher frequency of Tregs and CD38+ Treg, MHC class I gene loss, target antigen downregulation, and an abundance of CD8+ terminally exhausted cells (102, 108). In contrast, BsAb responders exhibit a biphasic immune response characterized by early T-cell receptor-independent expansion of CD8+ clones and a secondary T-cell receptor-driven response that can sustain anti-tumor immunity (102). CD4+ cells appear less involved in early clonal expansion but are likely to play a key supporting role in the BsAb response (113). As the T-cell response governs clinical outcomes following BsAbs, there is growing interest in augmenting this. One approach is designing BsAbs to avoid regulatory T-cells and preferentially engage CD8+ T cells (114). Combination strategies utilizing IMID, CELMoDs, and anti-CD38 antibodies are also being investigated (69, 115).

### Acquired resistance

**Figure 3:**
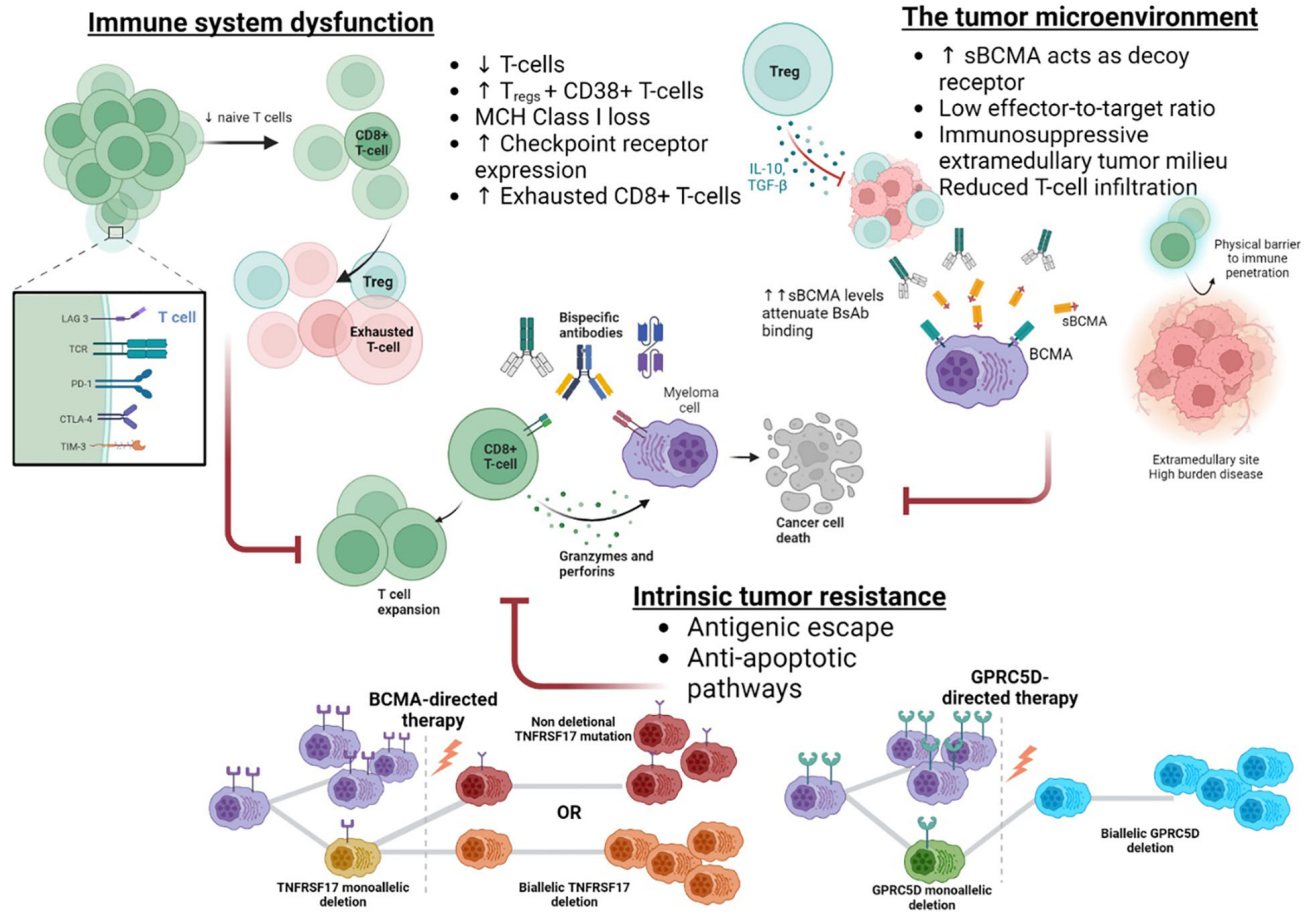
Resistance to bispecific antibody therapies.

Based on the application of similar immunotherapies in hematological diseases, immune system exhaustion and intrinsic plasma cell resistance via genomic or antigenic changes are the most likely causes of treatment failure, **Figure 3**. However, since re-treatment with BsAbs often produces sustained responses, immune system failure is unlikely to represent the primary form of treatment resistance (99). Antigenic drift is an established mechanism of immunotherapy evasion, most notably in B-cell acute lymphoblastic leukemia, where CD19 loss affects 40% of patients following anti-CD19 CAR T therapy (6). However, post BCMA-directed CAR T therapy, MM antigen loss is rare, affecting <5% of relapses, possibly because BCMA antigen loss requires a ‘double-hit’ event or since BCMA signalling is essential to plasma cell survival (103, 116, 117). Conversely, antigen loss at the time of relapse is nearly universal following GPRC5D-directed CAR T cells (118).

Nonetheless, genomic alterations in immunotherapy target genes exist in a moderate proportion of immunotherapy naïve patients; deletions at *TNFRSF17* and *GPRC5D* occur in 4-7% and 15% of individuals, respectively (116). These mutations predispose to antigen loss or alteration, and the subsequent application of potent immunotherapies creates the selective bottleneck required for the proliferation of these clones. Recently, antigenic changes have been identified as a major mechanism of treatment resistance following BsAb, affecting approximately 40% of BCMA- and 80-100% of GPRC5D-directed agents (101, 119). Eloquent single-cell analysis has shown that while biallelic *TNFRSF17* deletional events post-BsAb are rare, mutational events in the extracellular domain of BCMA in combination with monoallelic deletions are common (40%) and enable the retention of BCMA-mediated pro-survival signaling as well as escape from BCMA-directed BsAb binding and cytotoxicity (101). Importantly, the non-deletional mutations did not uniformly compromise BsAb binding, and some agents maintain *in-vitro* BCMA-binding and cytotoxicity, likely secondary to differences in TCE valency or structural design. Comparable to the experience with GPRC5D-CAR T, deletional or mutational events commonly facilitate GPRC5D antigen loss post BsAb exposure in up to 80-100% of cases (101, 119). The convergent evolution of both BCMA- and GPRC5D-directed BsAbs to produce antigen escape is a testament to the potent selection pressure exerted by BsAbs. Furthermore, the prolonged pressure created by the repeated BsAb dosing compared to one-off CAR T administration differentially influences treatment resistance mechanisms.

## Optimal sequencing of BsAbs

BCMA-directed BsAbs and CAR T were the first cellular therapies to obtain FDA approval in MM, and both CAR-T products can now be used in earlier disease stages. Recently the FDA expanded cilta-cel’s approval for use in patients who have received at least one line of therapy, including a PI, an IMID and are refractory to lenalidomide and ide-cel’s approval for patients who have had two or more lines of therapy, including an IMID, PI and anti-CD38 monoclonal antibody. Non-BCMA-directed BsAbs entered the market later, and to date, only talquetamab has received FDA approval for patients who have received four or more prior lines of therapy (11, 31, 92).

Both real-world and trial data show that the first BCMA-directed treatment is the most effective; however, CAR T outcomes appear more significantly compromised with delayed use than with BsAbs (93–96). Compared to the unexposed CARTITUDE-1 cohort, patients with prior BCMA therapy enrolled in CARTITUDE-2C had substantially lower response rates (ORR 60% vs. 98%) and median PFS (9 vs. 35 months) (95, 97). Likewise, the US Myeloma CAR T cell consortium found prior BCMA exposure reduced the response rate (ORR 74% vs. 88%) and median PFS (3 vs. 9 months) of ide-cel (96). In contrast, durable responses are still seen in patients who receive BsAb therapy following CAR T for both BCMA-directed (93, 94, 98) and non-BCMA-directed agents (69, 98, 99). In MonumenTAL-1, talquetamab’s ORR was 75% in patients with prior BCMA CAR T with a median DOR of 12.3 months (99). Conversely, for patients with prior BCMA BsAb therapy, talquetamab’s ORR was 52%, which fell further to an ORR of 29% in patients who received a BsAb as the immediate prior line (99). Elranatamab achieved an ORR of 54% in patients with prior BCMA-directed therapy (CAR T or ADC) compared to 64% when all patients were considered (93). A large cohort of relapsed patients following BCMA-directed CAR T therapy (n=79) identified superior outcomes with BsAb salvage (both BCMA-directed and other) compared to conventional doublet, triplet or quadruplet combinations of IMID, anti-CD38 antibodies and PIs with an ORR of 75% vs. 32%, and median PFS of 9.1 vs. under 4.5-months (98), highlighting the feasibility BsAb salvage post-CAR T (98). A similar study including a modest cohort of patients who relapsed following BsAb therapy (both BCMA- and GPRC5D-directed) confirmed that salvage with BsAb or CAR T was superior to doublet, triplet, or chemotherapeutic approaches (100).

Possible explanations for the diminished efficacy of CAR T following BsAb therapy include the antigen escape and T-cell exhaustion (101, 102). The sustained longitudinal selection pressure created by BCMA- and GPRC5D-directed BsAbs promotes antigen escape, which occurs in up to 40% of BCMA- and 80% of GPRC5D-BsAbs recipients (101). An abundance of exhausted CD8+ T-cell clones in the bone marrow is associated with an increased likelihood of relapse post BsAbs, and leukapheresis and manufacturing using exhausted T cell is associated with inferior *in-vitro* CAR T activity (102, 120). Treatment-free periods may mitigate T-cell exhaustion (104), in MonumenTAL-1, the ORR of talquetamab was lower in patients who received BsAb therapy as the immediate prior line compared to any prior line, with an ORR of 29% vs. 61%. Likewise, amongst patients who had received prior BsAb therapy, the ORR was 63% if the interval between the last prior BsAb was ≥ 9 months but reduced to 46% if the treatment interval was less than 6 months (99).

In summary, where practical, CAR-T therapy is the most effective primary cellular therapy in MM, and BsAbs constitute an effective form of salvage post-CAR-T. While data is limited, targeting an alternate antigen may be preferable. CAR T and BsAbs are effective salvage options for patients relapsing following BsAb therapy. Where possible, periods off BsAb therapy are desirable to reduce T cell exhaustion and improve response rates.

## Mitigating toxicity with BsAbs

CRS and ICANS are less frequent and less severe with BsAbs than CAR-T. Using ramp-up dosing, and pre-emptive strategies such as tocilizumab and corticosteroids has reduced this further without impacting treatment response (121). Instead, the major drawback of BsAbs is their increased infection risk compared to conventional MM therapies. A combined analysis of over 1,100 patients receiving both BCMA- and non-BCMA-directed BsAb outlined the key infectious complications inherent to this class (122). Prevalent severe adverse events (CTCAE Grade≥3) include neutropenia (35%), infections (25%), and pneumonia (10%). Non-BCMA-directed BsAbs have lower rates of severe neutropenia (25% vs 39%) and infections (12% vs. 30%). Notably, atypical infections, including pneumocystis jirovecii pneumonia (PJP, all grades 4%), cytomegalovirus (CMV, all grades 8%), candida esophagitis, and ophthalmic herpes simplex were all reported at higher incidences than is seen with other MM therapy. Lastly, approximately 75% of BsAb patients experience hypogammaglobulinemia (serum IgG level ≤400mg/dL), associated with an increased risk of infection with encapsulated bacteria (122).

The profound impact of infections in BsAb therapy has led to the publication of two expert consensus statements (123, 124). Common elements include universal prophylaxis against *pneumocystis jirovecii* with trimethoprim-sulfamethoxazole or an alternative agent, universal prophylaxis for varicella zoster and herpes simplex viruses with valacyclovir or acyclovir, immunoglobulin replacement for recurrent bacterial infections regardless of IgG level or for hypogammaglobulinemia (≤400mg/dL), screening for hepatitis B reactivation before treatment and the use of granulocyte-colony stimulating factor for patients with grade≥3 neutropenia.

Besides prophylactic measures, modifying the maintenance schedule of BsAb administration significantly influences infectious and myelosuppressive side effects. Switching to a biweekly schedule with elranatamab in magnetisMM-3 maintained treatment responses and reduced the incidence of grade 3-4 adverse events from 59% to 47%, particularly infections and myelosuppression (10). Equally, changing to a biweekly schedule of teclistamab does not appear to compromise efficacy, and a phase 2 trial of fixed duration therapy in responding patients is underway (NCT05932680), to mitigate T cell exhaustion (90). Likewise, talquetamab dosed biweekly compared to a weekly schedule was associated with a lower rate of infectious complications (34% vs. 47%) (11). Of note, the BCMA-directed bispecific antibodies delivered with less frequent dosing schedules, such as alnuctumab and ABBV-383, have reported lower rates of infection in their phase 1 trials, with Grade 3+ infection rates of 10% and 25%, respectively (25, 38).

## Future directions

TCE BsAbs have become integral to managing R/R MM, however, there is a high attrition rate with each additional line of therapy, particularly in older and comorbid patients, with less than 15% reaching the 4^th^ line (125). This suggests that these effective treatments should be utilized up front. Furthermore, therapies relying on endogenous T-cell function might be less effective if reserved for later lines due to T-cell exhaustion. Considering their efficacy and tolerability in older and frail patients, BsAbs could potentially have a role in the upfront treatment of patients deemed ineligible for conventional regimens. Numerous trials have been designed to assess the efficacy and safety of BsAbs as adjuncts to standard-of-care regimens in the first-line setting for transplant-eligible and ineligible patients.

Finally, the optimal schedule and duration of treatment with BsAbs have not yet been established. Currently, BsAbs are administered continuously until disease progression or intolerance. T-cell exhaustion induced by ongoing stimulation has been reported as one of the resistance mechanisms to T-cell-directing therapies (104). Thus, strategies to overcome T-cell exhaustion are currently being explored. Among those are treatment-free intervals and extended dosing schedules (126), a combination of BsAbs and immune checkpoint inhibitors, generation of trispecific antibodies that target PD-L1 (127), and concurrent treatment with low-dose cyclophosphamide, which improves effector T-cell function by depleting regulatory T-cells (128).

## Conclusions

BsAbs have transformed the treatment landscape of R/R MM, offering hope to patients with otherwise limited treatment options. The manufacturing techniques for BsAbs have evolved significantly over the years to enhance their pharmacokinetic and pharmacodynamic properties, and efforts are ongoing to improve effectiveness further, reduce toxicity, and allow more convenient dosing. Despite considerable progress in understanding resistance mechanisms, much remains to be learned. Preliminary data on combination strategies show great promise, particularly in patients with EMD. Several clinical trials are currently underway to evaluate new combinations at various disease stages. BsAbs have great potential for advancing to earlier lines of therapy in the upcoming years. As research progresses and more sophisticated constructs are being developed, efforts must be geared towards expanding access to these therapies and ensuring diverse patient representation and inclusion of older patients in clinical trials evaluating these and other novel therapies.
